# Phthalate Metabolites in Maternal Urine and Breast Milk After Very Preterm Birth: Matrix Concordance

**DOI:** 10.3390/toxics14020141

**Published:** 2026-01-30

**Authors:** Esin Okman, Sıddika Songül Yalçın, Deniz Arca Çakır, Fuat Emre Canpolat, Suzan Yalçın, Pınar Erkekoğlu

**Affiliations:** 1 Department of Pediatrics, Bilkent City Hospital, Ankara 06800, Türkiye; 2 Department of Social Pediatrics, Institute of Child Health, Hacettepe University, Ankara 06230, Türkiye; 3 Division of Social Pediatrics, Department of Pediatrics, Faculty of Medicine, Hacettepe University, Ankara 06230, Türkiye; 4 Department of Vaccine Technology, Vaccine Institute, Hacettepe University, Ankara 06100, Türkiye; denizcakir@hacettepe.edu.tr (D.A.Ç.); erkekp@hacettepe.edu.tr (P.E.); 5 Department of Neonatology, Bilkent City Hospital, University of Health Sciences, Ankara 06800, Türkiye; femrecan@gmail.com; 6 Department of Food Hygiene and Technology, Faculty of Veterinary Medicine, Selçuk University, Konya 42003, Türkiye; syalcin@selcuk.edu.tr; 7 Department of Pharmaceutical Toxicology, Faculty of Pharmacy, Hacettepe University, Ankara 06100, Türkiye

**Keywords:** endocrine disruptors, phthalates, environmental pollutants, breast milk, maternal factors

## Abstract

Background: Exposure to environmental pollutants, especially endocrine-disrupting chemicals, disproportionately affects vulnerable populations like pregnant women, lactating mothers, and preterm infants. This study aimed to assess the detection patterns of DiNP-, DEP-, and DEHP-related metabolites in maternal urine and breast milk, examine agreement between matrices, and explore maternal factors associated with phthalate exposure. Methods: Fifty-five mothers who delivered at ≤32 gestational weeks and whose infants were hospitalized in the Neonatal Intensive Care Unit (NICU) were enrolled. Breast milk and urine samples were analyzed using a validated isotope-dilution LC–MS/MS method. Urinary phthalate metabolite concentrations were adjusted for specific gravity. Linear mixed-effects models with a random intercept for mother were used to examine associations between urinary and breast milk phthalate metabolite concentrations, assess temporal changes, and evaluate the influence of breast milk lipid content. Results: DEHP and DiNP metabolites were detected in nearly all maternal urine samples. Breast milk contained predominantly primary metabolites (MEHP and MiNP), while secondary oxidative metabolites were rarely detected. Urine concentrations consistently exceeded breast milk concentrations. Urinary and breast milk phthalate concentrations were not correlated across sampling periods, indicating limited matrix concordance. Conclusions: Mothers of very preterm infants experience sustained phthalate exposure in the postpartum period; however, limited metabolite transfer to breast milk indicates that maternal urine remains the preferred biomonitoring matrix for assessing systemic phthalate exposure. Breast milk phthalate profiles exhibit compound-specific temporal changes and appear largely independent of concurrent urinary exposure biomarkers.

## 1. Introduction

Phthalates are a widely used class of industrial plasticizers added to polyvinyl chloride (PVC) and other polymers to improve flexibility and durability. They are commonly found in medical devices, food packaging, personal care products, household materials, and numerous consumer goods, resulting in ubiquitous human exposure through ingestion, inhalation, and dermal contact. Because phthalates are not chemically bound to plastics, they readily leach into the environment and biological systems, leading to continuous low-level exposure throughout daily life [1,2,3,4]. Phthalates are well-established endocrine-disrupting chemicals that can interfere with hormonal signaling pathways involved in reproduction, metabolism, and neurodevelopment, particularly during critical windows of susceptibility [4]. Human exposure begins early in life, raising particular concern for pregnant and lactating women, who represent physiologically vulnerable populations. Despite increasing scientific evidence, awareness of phthalate exposure and its potential health implications remains limited among certain high-risk groups, suggesting ongoing and possibly unrecognized exposure during sensitive life stages [5,6].

Due to their short biological half-lives, phthalates are rapidly metabolized into monoesters and oxidative metabolites, and human exposure is most commonly assessed using urinary biomarkers [4,7]. While urine is considered the standard matrix for evaluating recent systemic exposure, breast milk has gained attention as a relevant biological matrix for assessing infant exposure during the neonatal period—a critical window for growth and development. Breast milk is the optimal nutritional source for infants and provides essential immunological, metabolic, and developmental benefits, particularly for infants hospitalized in the neonatal intensive care unit (NICU) [8,9,10,11]. At the same time, breast milk may act as a potential route of transfer for certain environmental contaminants, including endocrine-disrupting chemicals, from mother to infant [12,13,14,15,16]. Although breast milk concentrations may not fully reflect the total magnitude of maternal exposure, they provide valuable information about the environmental context in which the mother resides and the potential exposure milieu relevant to the breastfeeding infant [17,18]. Owing to the ease and non-invasiveness of sample collection, breast milk may also serve as a practical matrix for longitudinal monitoring studies. However, reliance on a single biomonitoring matrix can impose important limitations due to differences in toxicokinetics and biodistribution [19]. Existing studies suggest that mammary transfer of phthalates may be limited and compound-specific, and findings regarding metabolite profiles and concordance between urine and breast milk remain inconsistent [14,15].

Importantly, most previous investigations of phthalate metabolites in breast milk have focused on mothers of term infants and often relied on a single biological matrix. Only a limited number of studies have simultaneously measured phthalate metabolites in paired maternal urine and breast milk samples [7,14]. Consequently, data remain scarce regarding systemic exposure, matrix-specific metabolite patterns, and mammary transfer in mothers of very preterm infants during the early postnatal period—a population with heightened vulnerability due to medical interventions, NICU-related exposures, and altered physiological states.

Therefore, the primary aim of this study was to investigate the presence, concentration levels, and detection frequencies of major phthalate metabolites related to diisononyl phthalate (DiNP), diethyl phthalate (DEP), and di(2-ethylhexyl) phthalate (DEHP) in two biological matrices—maternal urine and breast milk—collected during the early postnatal period from mothers of very preterm infants born before 32 weeks of gestation. Secondary objectives were to evaluate the concordance between urinary and breast milk phthalate metabolites, to assess temporal changes in breast milk concentrations, and to explore maternal clinical and obstetric characteristics associated with phthalate exposure and matrix-specific accumulation patterns.

We hypothesized that (i) phthalate metabolite concentrations and detection frequencies would differ between urine and breast milk due to matrix-specific metabolic and transfer mechanisms; (ii) breast milk phthalate metabolite levels would exhibit temporal changes during the early postnatal period; and (iii) maternal clinical and obstetric factors would be associated with variability in phthalate metabolite concentrations and profiles.

## 2. Materials and Methods

### 2.1. Study Group

The present study comprised mothers aged 19–45 who gave birth at 32 weeks of gestation or less and their infants were admitted to the NICU. Mothers who did not sign the informed consent form or mothers of the infants with major congenital anomalies were excluded from the study. The study protocol was approved by the Ethics Committee of Bilkent City Hospital, and informed consent was obtained from the participants.

The demographic data of the mothers were recorded from patient files and medical records. Maternal variables included gestational age at delivery (weeks), pre-pregnancy body mass index (BMI, kg/m^2^), weight gain during pregnancy (kg), and categorized weight gain. Maternal weight gain during pregnancy was classified in three groups as low, normal and high according to the gestational week and initial body mass indices of the mothers. Maternal gestational weight gain was classified as low, adequate, or excessive according to the Institute of Medicine (IOM) recommendations, taking into account pre-pregnancy BMI [20,21,22,23]. Additional maternal and pregnancy-related data comprised multiple pregnancies, in vitro fertilization (IVF) pregnancies, antenatal corticosteroid administration (complete/partial/none), gestational diabetes, hypothyroidism, pre-eclampsia, and medication use during pregnancy (aspirin, enoxaparin, α-methyldopa). Maternal hypothyroidism was defined as a pre-existing or pregnancy-diagnosed clinical condition requiring levothyroxine treatment. Thyroid function tests (TSH and free thyroxine [fT4]) measured at the time of delivery were within the reference range in all affected mothers. Gestational diabetes mellitus was diagnosed according to standard obstetric screening protocols applied during pregnancy. Mode of delivery was also recorded as Cesarean section or vaginal delivery.

### 2.2. Sample Size Justification Based on Detection Frequency

Assuming a conservative expected detection frequency of 50%, a 95% confidence level (Z = 1.96), and a tolerable absolute error of approximately 0.13, the minimum required sample size is approximately 55 participants. Therefore, the inclusion of 55 mothers in the present study is sufficient to estimate the detection frequencies of DiNP, DEP, and DEHP metabolites in both matrices with acceptable precision.

### 2.3. Sample Collection and Analysis of Phthalate Metabolites

Between June 2023 and January 2024, breast milk and maternal urine samples were collected into glass vials in the NICU of Bilkent City Hospital. Breast milk samples were collected by manual expression directly into vials and kept at −20 °C. Maternal urine samples were collected into phthalate-free glass containers during routine clinical care and stored at −20 °C until analysis. The samples were then transferred to Hacettepe University, Faculty of Pharmacy, Department of Toxicology, for analysis of phthalate metabolites.

#### 2.3.1. Chemicals, Reagents, and Target Analytes

All chemicals were of analytical or HPLC grade. All chemicals were purchased from Merck (Darmstadt, Germany). Glucuronidase/arylsulfatase enzyme (from *Helix pomatia*) was obtained from Roche (Mannheim, Germany). Solid-phase extraction (SPE) cartridges were Oasis HLB (Waters, Milford, MA, USA). Millipore Synthesis A10 system (Billerica, MA, USA) was used to clean the Milli-Q. Zorbax Eclipse XDB-C18 (2.1 × 150 mm, 3.5 µm) was from Agilent (Santa Clara, CA, USA). All metabolites and their isotopes were obtained from either Cambridge Isotope Laboratories (Tewksbury, MA, USA) or LGC Standards (Wesel, Germany).

Eight phthalates, which are primary and secondary metabolites of DEHP, DEP, and DiNP were investigated in breast milk and urine samples: Mono-(2-ethylhexyl) phthalate (MEHP), Mono-(2-ethyl-5-hydroxyhexyl) phthalate (MEHHP), Mono-(2-ethyl-5-oxohexyl) phthalate (MEOHP), Mono-(2-ethyl-5-carboxypentyl) phthalate (MECPP), Monoethyl phthalate (MEP), Monoisononyl phthalate (MiNP), Mono-carboxy-isooctyl phthalate (MCiOP), Mono-oxo-isononyl phthalate (MOiNP). Native phthalate monoester standards, including DEHP, DEP, DiNP, MEHP, MEHHP, MEOHP, MECPP, MEP, MiNP, MCiOP) MOiNP, as well as their corresponding stable-isotope-labeled internal standards, were purchased from either Cambridge Isotope Laboratories (Tewksbury, MA, USA) or LGC Standards (Wesel, Germany).

#### 2.3.2. Contamination Control and Quality Assurance

To minimize external contamination and prevent contact with plastic materials throughout the study, all glassware and laboratory materials were thoroughly decontaminated and silanized prior to sample collection and analysis. Glassware was solvent-rinsed by immersion in tetrahydrofuran: n-hexane (50:50, *v*/*v*) for 2 h, dried in an incubator for 2 h, and subsequently heated to 400 °C for 4 h in a tube dry block heater to ensure complete deplasticization.

All chemicals, solutions, and laboratory wares were screened for phthalate contamination before use. Procedural blanks consisting of ultrapure water were included in each analytical batch at a frequency of approximately one blank per 10 samples and processed through the entire analytical workflow. No phthalate metabolites were detected above the limits of detection (LOD) in any procedural blank; therefore, blank subtraction was not applied. Quantification relied on isotopically labeled internal standards for all analytes to correct for matrix effects and analytical losses. Internal standards were spiked at a concentration of 20 ng/mL to correct for matrix effects and analytical variability.

#### 2.3.3. Sample Preparation

Sample preparation included enzymatic deconjugation with β-glucuronidase/arylsulfatase from *Helix pomatia* at 37 °C for 1 h to hydrolyze conjugated phthalate metabolites. This was followed by solid-phase extraction (SPE) and quantitative analysis of the released monoesters by an isotope-dilution high-performance liquid chromatography–tandem mass spectrometry (LC–MS/MS) method. Immediately after sample aliquoting, isotope-labeled internal standards were added to each sample before β-glucuronidase treatment and solid-phase extraction, thereby enabling full isotope-dilution quantification across all analytical steps.

After deconjugation, the breast milk and urine samples were processed separately before SPE. For breast milk, a protein precipitation step was performed: 1 mL of milk was mixed with two volumes of cold acetonitrile–methanol (both are HPLC grade and from Sigma-Aldrich, Mannheim, Germany), vortexed for 30 s, and then centrifuged at 4000× *g* for 10–15 min at 4 °C. The clear supernatant was carefully decanted, avoiding the fat layer, into a new silanized tube. Urine samples (1 mL) were centrifuged at 4000× *g* for 10–15 min (4 °C) to remove particulates, and the supernatant was transferred to a clean silanized vial.

The resulting supernatants were adjusted to pH 6–7 and loaded slowly onto Oasis HLB SPE cartridges (Waters, Milford, MA, USA) at a dropwise flow to maximize analyte retention. Each cartridge was washed with 1 mL of ultrapure water (to remove salts and polar interferences), followed by 1 mL of 40% methanol–water (*v*/*v*) (to remove residual proteins and weakly bound matrix components). Phthalate monoesters were then eluted with 1 mL of methanol, followed by 1 mL of acetonitrile to ensure complete recovery of more hydrophobic analytes. The combined eluates were collected in silanized glass tubes, evaporated to dryness under a gentle stream of nitrogen at 40 °C, and stored at −20 °C until analysis. Prior to LC–MS/MS analysis, dried extracts were reconstituted in 100 µL of 10% methanol–water (*v*/*v*), vortex-mixed, and transferred to amber glass autosampler vials. Approximately every ten samples, a procedural blank (ultrapure water subjected to the entire extraction) was run to check for contamination, and a matrix spike (blank matrix spiked with 10 ng/mL mixed phthalate standards and internal standards) was processed in parallel to monitor extraction recovery. The recovery value range was 85–110% (Table S1).

#### 2.3.4. LC–MS/MS Analysis

Phthalate monoesters were analyzed using an Agilent 1100 HPLC system coupled to an API 4000 triple-quadrupole mass spectrometer (Applied Biosystems, Rockville, MD, USA). Chromatographic separation was achieved on a Zorbax Eclipse XDB-C18 reversed-phase column (2.1 × 150 mm, 3.5 µm; Agilent) at 40 °C, with a mobile phase of 0.1% acetic acid (HPLC grade, Sigma-Aldrich, Mannheim, Germany) in water (A) and methanol (B) at a flow rate of 0.2 mL/min. A linear gradient from 10% to 90% B over 10 min was employed, with a total run time of approximately 20 min and an injection volume of 10 µL. The mass spectrometer was operated in negative electrospray ionization (ESI–) mode (source temperature ~350 °C, capillary voltage −4500 V; nitrogen as nebulizer gas). Analyte detection was performed in multiple reaction monitoring (MRM) mode, with specific precursor → product ion transitions for each phthalate metabolite and its isotopically labeled internal standard (Table S1).

#### 2.3.5. Calibration, Quality Control, and Detection Limits

Calibration curves (0.5–200 ng/mL) were prepared (matrix-matched for urine) and fitted by weighted (1/x) linear regression. Quantification was carried out by isotope dilution, i.e., using a dedicated stable-isotope-labeled internal standard for each analyte, and for urine samples, the calibration standards were prepared in blank urine matrix. Samples were analyzed in four batches of ~21 samples each; each batch included calibration standards, procedural blanks, pooled matrix quality-control samples, and matrix-spiked controls fortified at 10 ng/mL. Extraction recoveries for all target metabolites were within the acceptable range of 85–110%, averaging approximately 90%. The method limits of detection (LOD) and quantification (LOQ) for the phthalate metabolites were in the low ng/mL range: for urine, LODs ~0.13–1.01 ng/mL (LOQs ~0.28–2.11 ng/mL); for breast milk, LODs ~0.27–2.12 ng/mL (LOQs ~0.51–3.99 ng/mL) (Table 1). Relative Standard Deviations (RSDs) for the measured elements for ten replicates are given in Table 1.

#### 2.3.6. Data Processing and Expression

All phthalate metabolite concentrations were initially obtained in ng/mL and subsequently converted to molar units (nmol/L) using the molecular weight of each metabolite prior to statistical analyses. To estimate the overall exposure to different parent phthalates, the molar sums of their specific metabolites were calculated [4]. For DEHP, the total metabolite concentration (ΣDEHP) was calculated as the sum of the molar concentrations of MEHP, MEHHP, MEOHP, and MECPP:

ΣDEHP = [MEHP] + [MEHHP] + [MEOHP] + [MECPP]

For DiNP, the total metabolite concentration (ΣDiNP) was obtained by summing the molar concentrations of MiNP, MOiNP, and MCiOP:

ΣDiNP= [MiNP] + [MOiNP] + [MCiOP]

The percentages of primary monoesters were calculated as MEHP% = (MEHP/ΣDEHP) ×100 and MiNP% = (MiNP/ΣDiNP) ×100.

### 2.4. Urine Dilution Correction by Specific Gravity

Urinary dilution was corrected using specific gravity (SG), which is the preferred approach for pregnancy and postpartum biomonitoring studies. Specific gravity was measured in all urine samples using a digital refractometer (PAL-10S Urine Specific Gravity Meter, ATAGO Co., Ltd., Tokyo, Japan) at 20 °C. To account for inter-individual and temporal variation in urine concentration, all urinary phthalate metabolite concentrations were adjusted according to the Levine–Boeniger formula using a reference SG of 1.020, as follows:

The formula:

CSG = Craw × SGref − 1/SGsamp − 1

C-raw (measured urinary concentration, nmol/L)

CSG (the SG-adjusted concentration)

SG-sample (the specific gravity of the individual urine sample)

SGref = 1.020 (a default/reference value for pregnant and lactating mothers)

### 2.5. Fat Analysis in the Milk Samples

Milk fat was estimated using the “creamatocrit method”. Thawed breast milk samples were warmed to room temperature, vortex-mixed for homogenization, loaded into heparinized microhematocrit capillary tubes, sealed, and centrifuged by Haematokrit 210 microhematocrit centrifuge (Hettich Zentrifugen, Tuttlingen, Germany) for 10–15 min at 12,000 rpm. Creamatocrit (%) was calculated as the ratio of the cream layer height to the total milk column height ×100. Where required, milk concentrations were additionally expressed per gram lipid using fat content derived from creamatocrit. Measurements were made in triplicate, and the mean fat value was used in the calculations.

### 2.6. Statistical Analysis

Statistical analyses were performed using IBM SPSS Statistics, version 23 (IBM Corp., Armonk, NY, USA).

Continuous variables were tested for distributional normality using the Shapiro–Wilk test and are presented as median and interquartile range (IQR) due to the non-normal distribution of phthalate concentrations. Concentrations below the limit of detection (LOD) were included as LOD/√2 for numerical analyses.

Comparisons of phthalate metabolite concentrations between maternal subgroups (e.g., gestational diabetes mellitus, hypothyroidism, medication use, mode of delivery, antenatal steroid exposure) were performed using Mann–Whitney U or Kruskal–Wallis tests, as appropriate, only when the smaller subgroup included at least 10 participants; otherwise, results were summarized descriptively due to limited statistical power.

Urinary phthalate metabolite concentrations were corrected for urine dilution using specific gravity measured at the time of collection, and SG-adjusted concentrations were subsequently log-transformed for analysis.

Breast milk phthalate metabolite concentrations were log-transformed.

Correlations between breast milk and maternal urinary phthalate concentrations across sampling days (Visit 1: First 24 h after delivery, Visit 2: Day 7, and Visit 3: Day 14–28) were evaluated using Spearman’s rank correlation coefficients (r_s_).

Linear mixed-effects models with a random intercept for mother were used to evaluate associations between maternal urinary phthalate metabolite concentrations and breast milk phthalate levels. Three hierarchical models were fitted: (1) a primary model including urinary phthalate concentration, sampling period, and breast milk lipid content; (2) an interaction model additionally including a urinary concentration × sampling period interaction term to assess time-dependent associations; and (3) a reduced model excluding urinary concentrations to evaluate temporal changes in breast milk concentrations. As a sensitivity analysis, breast milk phthalate concentrations were additionally expressed per gram of lipid (nmol/g lipid) and analyzed using the same mixed-effects modeling framework. These analyses were performed to assess the robustness of findings to lipid normalization, particularly for compounds with potential lipid partitioning.

Models were estimated using restricted maximum likelihood, and statistical significance was defined as *p* < 0.05.

## 3. Results

This study included 55 mothers who delivered at ≤32 weeks of gestation and whose infants were admitted to the NICU after birth. Ten mothers had twin births, and thus a total of 65 infants were included in the study. The mean maternal age was 30.7 ± 5.0 years, and the mean gestational age was 30.1 ± 1.9 weeks. Nearly half of the mothers had excessive gestational weight gain when compared to initial body mass indices. Cesarean section was the predominant mode of delivery (90.9%) (Table 2).

Detection frequencies and concentrations of phthalate metabolites in maternal urine across three study visits and in breast milk across two postpartum visits are presented in Table 3. Nearly all urine samples collected across the three time points contained detectable levels of DEHP and DiNP metabolites. In all visits, approximately 20% of ΣDEHP in urine consisted of the primary metabolite MEHP, while more than one-third of ΣDiNP consisted of MiNP (Table 3). Median urinary concentrations of individual DEHP metabolites (MEHP, MEHHP, MEOHP, and MECPP) and ∑DEHP showed no statistically significant differences between Visit 1 and Visit 2 or between Visit 1 and Visit 3. Similarly, urinary concentrations of DiNP-related metabolites (MiNP, MOiNP, and MCiOP) and ∑DiNP remained stable across visits. The relative contribution of primary DEHP metabolites, expressed as the MEHP/∑DEHP ratio, did not differ significantly across visits. In contrast, the proportion of MiNP relative to ∑DiNP in urine (MiNP/∑DiNP, %) showed a significant decrease over time, with lower median values observed at later visits (*p* = 0.009 for Visit 1 vs. Visit 2 and *p* = 0.005 for Visit 1 vs. Visit 3). In breast milk, detection frequencies were substantially lower than in urine and varied by metabolite class. At Visit 2, MEHP was detected in 38 of 55 breast milk samples (69.1%). All DEHP metabolites identified in breast milk were exclusively the primary metabolite MEHP, with no secondary metabolites detected. At the same visit, 41 of the 55 breast milk samples (74.5%) contained at least one of the three measured DiNP metabolites. In both sampling periods in which breast milk was analyzed, the primary metabolite MiNP accounted for more than half of ΣDiNP concentrations. Median breast milk MEHP concentrations increased significantly from Visit 2 to Visit 3 (*p* < 0.001). In contrast, breast milk concentrations of MiNP and ∑DiNP, as well as the MiNP/∑DiNP ratio, did not differ significantly between visits (Table 3).

Moderate to high positive correlations were observed between urinary phthalate metabolite concentrations measured at the three sampling points (Visit 1, Visit 2, and Visit 3). In particular, metabolites such as MiNP, MOiNP, MCiOP, and ∑DiNP demonstrated strong correlations across repeated urine measurements (r_s_ ranging from 0.52 to 0.92, *p* < 0.01). Similarly, DEHP metabolites (MEHP, MEHHP, MEOHP, and ∑DEHP) showed consistent within-subject correlations across time, indicating stable exposure patterns throughout the post neonatal period (Table 4). Strong correlations were observed between urinary phthalate metabolite concentrations across all sampling periods, indicating stable individual exposure ranking over time. In contrast, correlations between urinary and breast milk concentrations were weak and inconsistent. A modest positive correlation was observed between urinary and breast milk MiNP concentrations at the second visit, whereas a negative correlation was observed for MCiOP at the third visit (Table 4).

Maternal demographic and obstetric factors—including multiple gestation, conception via gravida status, preeclampsia, use of medications, mode of delivery, pre-pregnancy BMI category, gestational weight gain, and antenatal steroid administration—were not associated with either breast milk or maternal urinary phthalate concentrations (Table S2).

Linear mixed-effects models were used to examine associations between maternal urinary phthalate metabolites and breast milk concentrations across two sampling periods, accounting for repeated measurements within mothers. Urinary SG-adjusted phthalate metabolite concentrations were not significantly associated with breast milk concentrations of MEHP, MiNP, total DiNP, or the ratio of primary to total DiNP metabolites. Furthermore, no significant interactions between urinary concentrations and sampling period were observed, indicating that urinary–milk associations did not vary over time. In contrast, breast milk MEHP concentrations were significantly lower during the second visit compared with the third visit, independent of urinary MEHP levels and breast milk lipid content. No significant temporal changes were observed for MiNP, total DiNP, or the primary-to-total DiNP ratio. Breast milk lipid content was not independently associated with any phthalate metabolite outcome (Table 5).

In sensitivity analyses using lipid-normalized breast milk concentrations (nmol/g lipid), the results were consistent with the primary analyses. Breast milk MEHP concentrations remained significantly lower at the later sampling period, whereas no temporal changes or associations with urinary biomarkers were observed for MiNP. No significant urine-by-period interactions were detected (Table 6).

## 4. Discussion

Our study showed that urinary concentrations are consistently higher than those in breast milk across all phthalate metabolites measured, demonstrating low mammary transfer of phthalate metabolites. The presence of a proportional bias indicates that higher maternal exposures do not proportionally increase breast milk levels, suggesting metabolic or physiological barriers. This suggests a limited transfer of phthalates or their metabolites from maternal circulation to breast milk, likely due to low diffusion to mammary tissue, rapid renal elimination and/or the low lipid solubility of these specific metabolites.

A number of studies have demonstrated that the primary metabolite, MEHP, is present in breast milk; secondary DEHP metabolites are excreted into milk in either negligible or limited amounts [12,13,24]. Compared with previous studies, our results confirm the consistent presence of MEHP as the predominant DEHP metabolite in breast milk; no secondary metabolites of DEHP, including MEHHP, MEOHP and MECPP, were detected in breast milk in our study. Among DEHP metabolites, MEHP is relatively more lipophilic than its oxidative secondary metabolites, while secondary metabolites are more hydrophilic; this could be the reason we did not detect secondary metabolites in breast milk, even though we detected them in urine [12,14].

It is noteworthy that our measured MEHP levels in breast milk were substantially lower than most of the earlier studies (even if the studies used different units, comparisons were made after the units were converted to nmol/L). In a BAMBI study from Germany, the MEHP concentration was ~8.3 nmol/L, and breast milk samples were collected at 4–8 weeks postpartum [12]. In a Korean study, it was ~7.5 (sampling time was 1 month postpartum) and ~6.2 nmol/L (and hindmilk was collected from primiparous mothers attending breastfeeding clinics during lactation), respectively [13,24], and in a study from China, it was ~8.1 nmol/L, with donor milk samples being collected 3–4 weeks after delivery [15]. It is clear that these levels are 3–4 times higher than our MEHP levels. Several factors may contribute to this difference. First of all, the timing of the samplings is clearly different between studies, which would result in distinct milk lipid content. Our samples were obtained in the early postnatal period, when milk‘s lipid content is typically lower. Second, all of these earlier studies analyzed phthalates only in breast milk, unlike our study, which examined both milk and urinary levels of phthalates, allowing us to distinguish low mammary transfer and genuinely low systemic exposure. Finally, environmental and product-related phthalate exposure can be different among countries due to regulatory restrictions.

Notably, DiNP-related metabolites in breast milk were frequently detected in our cohort, whereas most earlier studies either did not analyze DiNP metabolites or reported non-detectable levels, highlighting population and time-specific exposure patterns [15,16,17,18,19]. As with DEHP metabolites, we found that the primary metabolite MiNP is predominantly transferred to breast milk from DiNP metabolites. Experimental and human biomonitoring data indicate that secondary oxidative metabolites of DiNP (including MOiNP and MCiOP) are more polar and are predominantly eliminated via urine, representing the most sensitive biomarkers of exposure. In contrast, breast milk has been described as a conditional biomonitoring matrix rather than a primary route of excretion. Even though we observed secondary DiNP metabolites in urine frequently, the lack of these metabolites in breast milk likely indicates that these hydrophilic metabolites are preferentially eliminated by the kidneys, as in DEHP metabolites.

The strong correlations observed across urinary samples over time suggest persistent and stable maternal phthalate exposure. However, the lack of consistent correlations between urinary and breast milk concentrations highlights the distinct biological nature of these matrices. Breast milk represents a separate compartment influenced by lipid partitioning, mammary gland transport, and potential local metabolism. The transient association observed for MiNP early postpartum may reflect limited transfer of primary DiNP metabolites, whereas the inverse association observed for MCiOP later postpartum suggests restricted or regulated transfer of secondary oxidative metabolites into breast milk as lactation progresses.

We detected no association between maternal obstetric factors and maternal phthalate exposure.

This study demonstrates compound-specific temporal patterns of phthalate metabolites in breast milk and highlights a limited correspondence between urinary biomarkers and breast milk concentrations. While urinary phthalate metabolite levels did not predict breast milk concentrations for any compound examined, breast milk MEHP concentrations increased significantly over time. Importantly, these temporal changes were observed independently of breast milk lipid content, which was not significantly associated with breast milk phthalate concentrations across models.

Urinary phthalate metabolites primarily reflect short-term exposure, whereas breast milk represents a distinct biological compartment influenced by lipid partitioning, mammary gland transport, and metabolic processing. The lack of a strong association between milk lipid content and phthalate concentrations suggests that factors beyond simple lipid solubility, such as selective transport or local metabolism within the mammary gland, may play an important role. The observed temporal increase in MEHP may reflect changes in maternal metabolism or lactational physiology, whereas the stability of MiNP and DiNP-related measures suggests compound-specific transfer or retention mechanisms. Notably, the ratio of primary DiNP metabolites to total DiNP in breast milk remained stable over time and was independent of both urinary ratios and milk lipid content, suggesting a regulated and consistent metabolic profile within breast milk. These findings underscore the importance of directly measuring breast milk concentrations when assessing infant exposure to phthalates, as urinary biomarkers—even when adjusted for urine dilution—may not adequately capture exposure through lactation.

This study has several limitations that should be acknowledged. Although repeated measurements were available for a subset of participants, the observational design precludes causal inference. Sampling was restricted to discrete postpartum time points and therefore may not fully capture the short biological half-lives of phthalates or their substantial day-to-day variability in exposure. In addition, small sample sizes in certain maternal subgroups limited statistical power and precluded robust or reliable subgroup analyses, necessitating a cautious interpretation of exploratory findings.

These limitations should be interpreted in light of several important strengths of the present study. The paired maternal urine–breast milk design with repeated measurements provides a robust framework to examine matrix-specific phthalate profiles during an early and highly vulnerable postnatal period in mothers of very preterm infants—an understudied population with distinct exposure pathways. The consistent lack of association between urinary and breast milk phthalate concentrations across sampling periods, together with longitudinal linear mixed-effects modeling, strengthens the conclusion that maternal urine represents the more reliable biomonitoring matrix for assessing systemic phthalate exposure. Methodologically, the study benefited from rigorous contamination control procedures and a validated isotope-dilution LC–MS/MS analytical approach with low limits of detection, ensuring high analytical sensitivity and specificity. The use of linear mixed-effects models enabled efficient use of repeated measures while appropriately accounting for within-mother correlation and incomplete follow-up. In addition, urinary phthalate concentrations were adjusted for specific gravity, and breast milk lipid content was directly measured and incorporated into both primary and sensitivity analyses, enhancing comparability across matrices and strengthening internal validity. Finally, the compound-specific findings are supported by biologically plausible mechanisms, including differences in lipophilicity, mammary transfer, and metabolic processing between primary monoesters (e.g., MEHP) and secondary oxidative metabolites of DEHP and DiNP. The simultaneous assessment of maternal urine and breast milk across multiple sampling periods therefore provides novel and mechanistically informed insight into matrix-specific phthalate exposure patterns following very preterm birth.

## 5. Conclusions

In this study of mothers of very preterm infants, phthalate metabolite concentrations were consistently higher in maternal urine than in breast milk across sampling periods, indicating limited transfer of most metabolites into breast milk during the early postpartum period. The lack of concordance between urinary and breast milk concentrations suggests that maternal urine represents a more reliable biomonitoring matrix for assessing systemic phthalate exposure, whereas breast milk reflects a distinct biological compartment influenced by compound-specific transfer and retention processes. In breast milk phthalate profiles, a significant increase was observed for MEHP over time, while DiNP-related metabolites remained relatively stable. Together, these findings highlight the need for future longitudinal studies with larger sample sizes and more frequent sampling to further elucidate matrix-specific exposure dynamics and potential implications for maternal and infant health.

## Figures and Tables

**Table 1:** Limit of Detection (LOD) and Limit of Quantification (LOQ) for each biological matrix and Relative Standard Deviations (RSDs).

	Urine				Breast Milk			
Phthalate Metabolite	LOD (ng/mL)	LOQ (ng/mL)	Inter-Day RSD (%)	Intra-Day RSD (%)	LOD (ng/mL)	LOQ (ng/mL)	Inter-Day RSD (%)	Intra-Day RSD (%)
MEHP	0.13	0.28	4.54	5.51	0.27	0.51	2.43	3.60
MEHHP	0.87	1.81	5.45	5.47	1.56	2.88	4.09	5.52
MEOHP	0.59	1.13	2.36	4.18	1.02	2.07	2.29	3.51
MECPP	0.61	1.34	5.30	5.71	1.28	2.21	2.79	3.27
MEP	0.49	1.02	3.20	4.06	0.99	2.08	5.32	5.62
MiNP	1.01	2.11	3.83	4.49	2.12	3.99	6.72	5.10
MOiNP	0.31	0.66	3.35	4.54	0.61	1.19	2.57	3.43
MCiOP	0.56	1.18	4.75	5.61	1.06	2.17	3.39	4.53

**Table 2:** Demographic and clinical characteristics of participants.

Maternal Characteristics (*n* = 55)	Mean ± SD or *n* (%)
Maternal age (years)	30.7 ± 5.0
Gestational age (weeks)	30.1 ± 1.9
Maternal BMI (kg/m^2^)	25.6 ± 5.2
Weight gain during pregnancy (kg)	8.8 ± 6.9
Weight gain category (low/normal/high)	17 (30.9%)/13 (23.6%)/25 (45.5%)
Multiple pregnancy	10 (18.2%)
IVF pregnancy	6 (10.9%)
Antenatal steroid (complete/partial/none)	38 (69.1%)/12 (21.8%)/5 (9.1%)
Gestational diabetes	4 (7.3%)
Hypothyroidism	9 (16.4%)
Preeclampsia	17 (30.9%)
Aspirin use	12 (21.8%)
Enoxaparin use	8 (14.5%)
Methyldopa use	10 (18.2%)
Cesarean delivery	50 (90.9%)
Feeding in first 7 days, exclusive BM	41 (74.5%)
Urinary specific gravity	
Visit 1 (*n* = 55)	1.014 (1.009–1.021)
Visit 2 (*n* = 43)	1.014 (1.011–1.021)
Visit 3 (*n* = 29)	1.016 (1.010–1.019)
Breast milk lipid, g/L	
Visit 2 (*n* = 55)	32.5 (31.8–35.6)
Visit 3 (*n* = 32)	33.8 (32.6–35.6)

BMI: body mass index, IVF: in vitro fertilization, BM: breast milk. Data are presented as mean ± SD for continuous variables and *n* (%) for categorical variables.

**Table 3:** Maternal urinary and milk phthalate metabolite concentrations and detection frequencies on Visit 1, Visit 2 and Visit 3.

		Visit 1-MU 1 (*n* = 55)		Visit 2-MU 2 (*n* = 43)		Visit 3-MU 3 (*n* = 29)	*p* 1–2	*p* 1–3
Maternal Urine	DF, %	Median (25–75 *p*)	DF, %	Median (25–75 *p*)	DF, %	Median (25–75 *p*)		
MEHP, nmol/L-SG	100.0	21.9 (16.4–30.3)	100.0	25.6 (19.2–34)	100.0	26.6 (21.3–41.9)	0.192	0.274
MEHHP, nmol/L-SG	100.0	28.4 (20.1–36.5)	100.0	33.1 (24.5–44.6)	96.8	31 (27.2–50.6)	0.180	0.157
MEOHP, nmol/L-SG	100.0	29.7 (23.3–44.3)	100.0	32.4 (23.2–46.6)	96.8	37.3 (28.7–48.6)	0.447	0.165
MECPP, nmol/L-SG	100.0	33.3 (24–59.9)	95.3	36.4 (24.1–58.6)	93.5	36.7 (28.7–70.9)	0.461	0.163
MEP, nmol/L-SG	94.5	82.8 (60.2–127)	90.7	78.0 (63.5–114.0)	93.5	84.5 (68.4–138.2)	0.257	0.719
MiNP, nmol/L-SG	98.2	14.8 (9.5–22.9)	100.0	15.3 (11.1–23.2)	90.3	12.4 (7.8–25.3)	0.246	0.122
MOiNP, nmol/L-SG	89.1	12.0 (8.5–17.5)	93.0	13.1 (9.9–18.9)	90.3	15.3 (9.9–23.4)	0.763	0.387
MCiOP, nmol/L-SG	94.5	12.0 (8.0–17.9)	95.3	13.3 (9.6–17.1)	96.8	15.9 (9.6–21.0)	0.971	0.569
∑DEHP, nmol/L-SG	100.0	118 (87–170)	100.0	127 (96–187)	100.0	127 (112–213)	0.251	0.158
MEHP/∑DEHP, %		27.4 (19.5–37.5)		26.1 (19.9–39.1)		24.7 (20.3–37.4)	0.192	0.096
∑DiNP, nmol/L-SG	98.2	40.7 (25.4–57.2)	100.0	42.3 (30.9–52.3)	100.0	41.9 (33.3–64.6)	0.546	0.873
MiNP/∑DiNP, %		54.5 (41.6–84.6)		50.3 (37.3–77.8)		44.2 (33.2–74.2)	0.009	0.005
Maternal milk				Visit 2–MM 2 (*n* = 55)		Visit 3–MM 3 (*n* = 32)		*p* 2–3
MEHP, nmol/L			69.1	1.37 (<LOD-1.87)	90.6	1.78 (1.04–2.16)		<0.001
MiNP, nmol/L			70.9	7.98 (<LOD-9.79)	68.8	8.82 (<LOD-10.84)		0.162
MOiNP, nmol/L			41.8	<LOD (<LOD-6.88)	37.5	<LOD (<LOD-7.04)		
MCiOP, nmol/L			38.2	<LOD (<LOD-6.18)	28.1	<LOD (<LOD-5.72)		
∑DiNP, nmol/L			74.5	13.95 (0–21.68)	75.0	14.16 (1.84–20.77)		0.103
				(*n* = 41)		(*n* = 24)		
MiNP/∑DiNP, %				55.76 (38.73–100)		61.75 (39.26–100)		0.570

MU: maternal urine, MM: maternal milk. DF: detection frequency (% of samples ≥ LOD). ΣDEHP = molar sum of DEHP metabolites (MEHP, MEHHP, MEOHP, MECPP); ΣDiNP = molar sum of DiNP metabolites (MiNP, MOiNP, MCiOP); percent ratios (MEHP/ΣDEHP, MiNP/ΣDiNP) are given as percentages of the sum.

**Table 4:** Spearman correlation coefficients of urinary phthalate levels (nmol/L-SG) and breast milk phthalate levels (nmol/L) across sampling periods.

	MU1 & MU2	MU1 & MU3	MU2 & MU3	MU2 & MM2	MU3 & MM3
*n*	43	29	22	43	27
MEHP	0.679 **	0.524 **	0.677 **	−0.065	0.169
MEHHP	0.748 **	0.648 **	0.927 **		
MEOHP	0.789 **	0.672 **	0.873 **		
MECPP	0.777 **	0.510 **	0.630 **		
MEP	0.744 **	0.720 **	0.710 **		
MiNP	0.846 **	0.551 **	0.561 **	0.352 *	0.264
MOiNP	0.774 **	0.865 **	0.944 **	−0.052	−0.106
MCiOP	0.794 **	0.826 **	0.921 **	0.044	−0.413 *
∑DEHP	0.792 **	0.626 **	0.799 **		
MEHP/∑DEHP	0.744 **	0.536 **	0.666 **		
∑DiNP	0.816 **	0.892 **	0.894 **	0.184	0.016
*n*				32	
MiNP/∑DiNP	0.851 **	0.565 **	0. 602 **	0.379 *	0.183

MU: maternal urine, MM: maternal milk. Spearman correlation coefficients between maternal urinary phthalate metabolite concentrations measured at three different sampling periods (MU1, MU2, MU3) and between maternal urinary and breast milk phthalate concentrations (MM). Correlations were calculated using the non-parametric Spearman’s rank test. * Correlation is significant at the 0.05 level (2-tailed). ** Correlation is significant at the 0.01 level (2-tailed).

**Table 5:** Associations between maternal urinary phthalate metabolites and breast milk concentrations across sampling periods (linear mixed-effects models).

	MM logMEHP		MM logMiNP		MM log∑DiNP		MM log MiNP/∑DiNP	
Parameter	β (SE)	*p*	β (SE)	*p*	β (SE)	*p*	β (SE)	*p*
Model 1								
Urinary phthalate (SG-adjusted, log)	−0.36 (0.34)	0.293	−0.01 (0.29)	0.966	0.04 (0.12)	0.716	0.14 (0.12)	0.230
Period (Visit 2 vs. 3)	−0.28 (0.11)	0.017	0.02 (0.10)	0.851	−0.02 (0.02)	0.377	−0.01 (0.02)	0.363
Breast milk lipid (g/L)	0.04 (0.03)	0.186	−0.03 (0.04)	0.494	0.01 (0.01)	0.231	0.00 (0.01)	0.838
Model 2								
Urinary phthalate (SG-adjusted, log)	−0.41 (0.48)	0.402	−0.03 (0.33)	0.931	0.08 (0.13)	0.529	0.09 (0.13)	0.497
Period (Visit 2 vs. 3)	−0.39 (0.88)	0.661	−0.05 (0.47)	0.908	0.04 (0.10)	0.683	−0.16 (0.12)	0.231
Urin. phthalate × Period	0.07 (0.61)	0.903	0.06 (0.39)	0.871	−0.03 (0.06)	0.576	0.08 (0.07)	0.273
Breast milk lipid (g/L)	0.04 (0.03)	0.193	−0.03 (0.04)	0.522	0.01 (0.01)	0.184	−0.01 (0.01)	0.542
Model 3								
Period (Visit 2 vs. 3)	−0.29 (0.09)	0.003	−0.01 (0.07)	0.928	−0.02 (0.01)	0.233	−0.01 (0.01)	0.418
Breast milk lipid (g/L)	0.04 (0.03)	0.148	−0.03 (0.03)	0.388	0.01 (0.01)	0.183	0.00 (0.01)	0.900

MM: maternal milk.

**Table 6:** Sensitivity analyses using lipid-normalized breast milk phthalate metabolite concentrations (nmol/g lipid).

Outcome (log, nmol/g lipid)	Fixed Effect	Estimate (SE)	*p* Value
MEHP (lipid-normalized)	Urinary MEHP (SG-adjusted, log)	−0.17 (0.21)	0.424
	Sampling period (Visit 2 vs. 3)	−0.18 (0.07)	0.011
MEHP (lipid-normalized)	Urinary MEHP × Period	0.04 (0.36)	0.911
MiNP (lipid-normalized)	Urinary MiNP (SG-adjusted, log)	0.02 (0.21)	0.916
	Sampling period (Visit 2 vs. 3)	0.03 (0.06)	0.656
MiNP (lipid-normalized)	Urinary MiNP × Period	0.02 (0.28)	0.945

## Data Availability

Due to privacy-related reasons, the data presented in this study are only available on request from the corresponding author.

## Supplementary Materials

The following supporting information can be downloaded at: https://www.mdpi.com/article/10.3390/toxics14020141/s1, Table S1: Recovery rates (%) of phthalate metabolites in breast milk and urine and LC–MS/MS MRM transitions used for quantification. Table S2: Differences in Breast Milk and Maternal Urinary Phthalate Levels According to Maternal Clinical and Obstetric Characteristics.

## Abbreviations

The following abbreviations are used in this manuscript:

NICU	Neonatal Intensive Care Unit
DiNP	Diisononyl phthalate
DEP	Diethyl phthalate
DEHP	Di(2-ethylhexyl) phthalate
IVF	In vitro fertilization
MEHP	Mono-(2-ethylhexyl) phthalate
MEHHP	Mono-(2-ethyl-5-hydroxyhexyl) phthalate
MEOHP	Mono-(2-ethyl-5-oxohexyl) phthalate
MECPP	Mono-(2-ethyl-5-carboxypentyl) phthalate
MEP	Monoethyl phthalate
MiNP	Monoisononyl phthalate
MCiOP	Mono-carboxy-isooctyl phthalate
MOiNP	Mono-oxo-isononyl phthalate
SPE	Solid-phase extraction
LC–MS/MS	Liquid chromatography–tandem mass spectrometry
LOD	Limit of detection
