# Supplementary material for: Phthalate Metabolites in Maternal Urine and Breast Milk After Very Preterm Birth: Matrix Concordance
Source: Toxics. 2026 Jan 30;14(2):141. doi: 10.3390/toxics14020141 (PMC12944914; doi:10.3390/toxics14020141)
Supplement: Supplementary file 1 [file toxics-14-00141-s001.zip › Supplemenatry Table S1.pdf]

Table S1. Recovery values and MRM transitions of phthalate metabolites in breast milk and urine.

Recovery values for the phthalate metabolites (% ± SD) after SPE:

Milk:

- MEHP = 89.02 ± 3.27 %
- MEHHP = 89.19 ± 4.18%
- MEOHP = 90.03 ± 4.27%
- MECPP: 90.11 ± 3.88%
- MEP: 88.24 ± 5.12%
- MiNP: 90.33± 4.56%
- MOİNP = 92.28 ± 4.19%
- MCiOP = 90.27 ± 5.49%

Urine:

- MEHP = 90.71 ± 5.21 %
- MEHHP = 91.58 ± 4.88%
- MEOHP = 91.12 ± 5.38%
- MECPP: 92.14 ± 4.88%
- MEP: 90.81 ± 3.27%
- MiNP: 91.72± 4.01%
- MOİNP = 94.18 ± 6.16%
- MCiOP = 92.03 ± 7.14%

Specific m/z precursor → product ions:

MEHP: 277 → 134 m/z Quant. ion (m/z); 277 → 121 Qual. ion (m/z)

MEHHP: 293 → 121 m/z Quant. ion (m/z); 277 → 121 Qual. ion (m/z)

MEOHP: 291 → 121 Quant. ion (m/z); 277 → 121 Qual. ion (m/z)

MECPP: 163 → 121 m/z Quant. ion (m/z); 167 → 125 Qual. ion (m/z)

MEP: 193 → 121 m/z Quant. ion (m/z); 193 → 77 Qual. ion (m/z)

MiNP: 277 → 121 m/z Quant. ion (m/z); 281 → 125 Qual. ion (m/z)

MOiNP: 291→ 121 m/z Quant. ion (m/z); 295→ 125 Qual. ion (m/z)

MCiOP: 305→ 121 m/z Quant. ion (m/z); 309→ 125 Qual. ion (m/z)
