# Supplementary material for: Phthalate Metabolites in Maternal Urine and Breast Milk After Very Preterm Birth: Matrix Concordance
Source: Toxics. 2026 Jan 30;14(2):141. doi: 10.3390/toxics14020141 (PMC12944914; doi:10.3390/toxics14020141)
Supplement: Supplementary file 1 [file toxics-14-00141-s001.zip › Supplemenatry Table S2.pdf]

Table S2. Differences in Breast Milk and Maternal Urinary Phthalate Levels According to Maternal Clinical and Obstetric Characteristics

Test Statistics	Multiple pregnancy*	Gravida-Parity*	Preeclampsia*	ANS**	α-methyldopa*	Aspirin*	Weight Gain**	C/S*	Feeding of the infant**
**Maternal milk**									
MEHP2	0.921	0.747	0.760	0.401	0.715	0.983	0.337	0.744	0.883
MiNP2	0.365	0.841	0.803	0.899	0.877	0.256	0.247	0.988	0.328
MOiNP2	0.312	0.167	0.583	0.972	0.223	0.532	0.796	0.987	0.477
MCiOP2	0.216	0.673	0.610	0.644	0.194	0.333	0.939	0.615	0.433
∑DiNP2	0.605	0.703	0.451	0.938	0.367	0.886	0.461	0.871	0.212
MiNP/∑DiNP2	0.561	0.223	0.405	0.680	0.055	0.213	0.585	0.685	0.883
**Maternal urine.** nmol/L									
MEHP1	0.116	0.979	0.316	0.314	0.743	0.507	0.086	0.482	0.439
MEHHP1	0.187	0.686	0.199	0.028	0.230	0.959	0.294	0.918	0.946
MEOHP1	0.089	0.445	0.978	0.131	0.290	0.203	0.129	0.907	0.915
MECPP1	0.156	0.897	0.920	0.752	0.608	0.823	0.954	0.861	0.985
MEP1	0.810	0.864	0.450	0.877	0.513	0.476	0.932	0.758	0.678
MiNP1	0.089	0.850	0.402	0.602	0.222	0.078	0.815	0.815	0.862
MOiNP1	0.230	0.197	0.255	0.625	0.305	0.501	0.713	0.247	0.801
MCiOP1	0.359	0.636	0.402	0.753	0.930	0.625	0.654	0.538	0.602
∑DEHP1	0.222	0.630	0.771	0.561	0.359	0.967	0.864	0.838	0.847
MEHP /∑DEHP1	0.144	0.630	0.334	0.781	0.222	0.760	0.099	0.520	0.938
∑DiNP1	0.097	0.372	0.382	0.456	0.383	0.684	0.694	0.482	0.832
MiNP/∑DiNP1	0.285	0.235	0.820	0.845	0.825	0.063	0.320	0.199	0.969
**Maternal urine.** nmol/L-SG									
MEHP1	0.965	0.116	0.729	0.872	0.810	0.895	0.676	0.942	0.900
MEHHP1	0.948	0.354	0.122	0.591	0.743	0.935	0.754	0.682	0.728
MEOHP1	0.371	0.069	0.187	0.796	0.513	0.271	0.635	0.826	0.938
MECPP1	0.138	0.319	0.308	0.824	0.743	0.839	0.907	0.501	0.728
MEP1	0.662	0.419	0.105	0.828	0.965	0.839	0.786	0.639	0.817
MiNP1	0.585	0.295	0.089	0.931	0.266	0.403	0.780	0.826	0.885
MOiNP1	0.827	0.434	0.113	0.841	0.432	0.308	0.773	0.455	0.757
MCiOP1	0.853	0.311	0.226	0.955	0.965	0.210	0.893	0.473	0.582
∑DEHP1	0.432	0.164	0.196	0.892	0.631	0.555	0.820	0.619	0.938
MEHP /∑DEHP1	0.896	0.175	0.489	0.918	0.585	1.000	0.792	0.861	0.985
∑DiNP1	0.631	0.471	0.117	0.814	0.432	0.760	0.954	0.520	0.847
MiNP/∑DiNP1	0.841	0.056	0.179	0.937	0.862	0.803	0.545	0.521	0.890

*Mann Whitney U test; **Kruskal Wallis Test
